# Re-Creating Missing Population Baselines for Pacific Reef Sharks

**DOI:** 10.1111/j.1523-1739.2012.01835.x

**Published:** 2012-06

**Authors:** Marc O Nadon, Julia K Baum, Ivor D Williams, Jana M Mcpherson, Brian J Zgliczynski, Benjamin L Richards, Robert E Schroeder, Russell E Brainard

**Affiliations:** * Joint Institute for Marine and Atmospheric Research, University of Hawaii1000 Pope Road, Honolulu, HI 96822, U.S.A.; † Rosenstiel School of Marine and Atmospheric Science, University of Miami4600 Rickenbacker Causeway, Miami, FL 33149, U.S.A.; ‡ Pacific Islands Fisheries Science Center, NOAA Fisheries2570 Dole St., Honolulu, HI 96822, U.S.A.; ¶ National Center for Ecological Analysis and Synthesis (NCEAS), University of California Santa Barbara735 State Street, Suite 300, Santa Barbara, CA 93101, U.S.A.; †† Centre for Conservation Research, Calgary Zoological Society1300 Zoo Road NE, Calgary, Alberta T2E 7V6, Canada; ‡‡ Biological Sciences, Simon Fraser University8888 University Drive, Burnaby, BC V5A 1S6, Canada; §§ Center for Marine Biodiversity and Conservation, Scripps Institution of Oceanography, University of CaliforniaSan Diego, 9500 Gilman Drive, San Diego, CA 92093, U.S.A.; ¶¶ Pacific Islands Regional Office, NOAA Fisheries1601 Kapiolani Boulevard, Suite 11100, Honolulu, HI 96814, U.S.A.

**Keywords:** coral reefs, hierarchical model, marine macroecology, predators, species distribution modeling

## Abstract

**Abstract:**

Sharks and other large predators are scarce on most coral reefs, but studies of their historical ecology provide qualitative evidence that predators were once numerous in these ecosystems. Quantifying density of sharks in the absence of humans (baseline) is, however, hindered by a paucity of pertinent time-series data. Recently researchers have used underwater visual surveys, primarily of limited spatial extent or nonstandard design, to infer negative associations between reef shark abundance and human populations. We analyzed data from 1607 towed-diver surveys (>1 ha transects surveyed by observers towed behind a boat) conducted at 46 reefs in the central-western Pacific Ocean, reefs that included some of the world's most pristine coral reefs. Estimates of shark density from towed-diver surveys were substantially lower (<10%) than published estimates from surveys along small transects (<0.02 ha), which is not consistent with inverted biomass pyramids (predator biomass greater than prey biomass) reported by other researchers for pristine reefs. We examined the relation between the density of reef sharks observed in towed-diver surveys and human population in models that accounted for the influence of oceanic primary productivity, sea surface temperature, reef area, and reef physical complexity. We used these models to estimate the density of sharks in the absence of humans. Densities of gray reef sharks (*Carcharhinus amblyrhynchos*), whitetip reef sharks (*Triaenodon obesus*), and the group “all reef sharks” increased substantially as human population decreased and as primary productivity and minimum sea surface temperature (or reef area, which was highly correlated with temperature) increased. Simulated baseline densities of reef sharks under the absence of humans were 1.1–2.4/ha for the main Hawaiian Islands, 1.2–2.4/ha for inhabited islands of American Samoa, and 0.9–2.1/ha for inhabited islands in the Mariana Archipelago, which suggests that density of reef sharks has declined to 3–10% of baseline levels in these areas.

**Resumen:**

Los tiburones y otros depredadores mayores son escasos en la mayoría de los arrecifes de coral, pero estudios de su ecología histórica proporcionan evidencia cualitativa de que los depredadores una vez fueron numerosos en estos ecosistemas. Sin embargo, la cuantificación de la densidad de tiburones en ausencia de humanos (línea de base) es obstaculizada por la falta de datos de series de tiempo pertinentes. Recientemente, los investigadores han utilizado muestreos visuales submarinos, de extensión espacial limitada o de diseño no estándar, para inferir asociaciones negativas entre la abundancia de tiburones de arrecife y las poblaciones humanas. Analizamos datos de 1607 muestreos por remolque de buzos (transectos >1ha muestreados por observadores remolcados por una lancha) realizados en 46 arrecifes en el Océano Pacífico centro-occidental, arrecifes que incluyeron algunos de los más prístinos del mundo. Las estimaciones de densidad de tiburones fue sustancialmente menor (<10%) que estimaciones publicadas a partir de muestreos a lo largo de transectos pequeños (<0.02 ha), lo cual no es consistente con las pirámides de biomasa invertidas (la biomasa de depredadores es mayor que la biomasa de presas) reportadas para arrecifes prístinos por otros autores. Examinamos la relación entre la densidad de tiburones de arrecife observados en los muestreos por remolque de buzos y la población humana en modelos y consideramos la influencia de la productividad oceánica primaria, la temperatura de la superficie del mar, la superficie del arrecife y su complejidad física. Utilizamos estos modelos para estimar la densidad de tiburones en ausencia de humanos. Las densidades de *Carcharhinus amblyrhynchos, Triaenodon obesus* y el grupo de “tiburones estrictamente arrecifales” incrementó sustancialmente a medida que disminuyó la población humana y que incrementó la productividad primaria y la temperatura de la superficie del mar (o superficie del arrecife, que estaba altamente correlacionada con la temperatura. Las densidades basales simuladas de tiburones arrecifales en ausencia de humanos fueron 1.1–2.4/ha para las Islas Hawaianas, 1.2–2.4/ha en islas deshabitadas de Samoa Americana y 0.9–2.1/ha e islas deshabitadas del Archipiélago Mariana, lo que sugiere que la densidad de tiburones arrecifales ha declinado entre 3 -10% en relación con los niveles basales en esas áreas.

## Introduction

Sharks are high-level predators whose importance in oceanic ecosystems is increasingly recognized (Stevens et al. 2000; Myers et al. 2007; Ferretti et al. 2010). Over the last 4 decades many shark species have been heavily affected by the harvesting of shark fins (Clarke et al. 2006), fisheries bycatch (Mandelman et al. 2008), and recreational fishing (Fisher & Ditton 1993). Consequently, many sharks, particularly oceanic species, have been overexploited (Baum et al. 2003; Dulvy et al. 2008). The status of coral-reef associated sharks is less clear. Studies of the historical ecology of reefs suggest a widespread loss of large predators from these ecosystems (Jackson 2001; Pandolfi et al. 2003). Long-term time series data with which to quantitatively assess the status of reef sharks are lacking because in general reef sharks are not targeted in commercial fisheries and have been a low research priority (FAO 2008).

To gain insight into the current status of reef sharks, researchers have used nontraditional data sources and approaches to explore the influence of human populations on shark abundance. For example, Ward-Paige et al. (2010a) used shark sightings from roving surveys (nonstandard sampling area surveyed by free-swimming observers) and found that, with the exception of nurse sharks (*Ginglymostoma cirratum*), reef sharks are largely absent on Caribbean reefs and occur primarily where human population densities are low. Graham et al. (2010) inferred that reef sharks have declined by 90% at 3 atolls in the Chagos Archipelago (central Indian Ocean) from a comparison of shark sightings made by researchers conducting roving surveys at 5 points in time between 1975 and 2006. In the Pacific Ocean, results of belt-transect surveys (rectangular sampling area of fixed dimensions surveyed by observers swimming along a central transect line) showed significant differences in shark biomass between inhabited and remote reef areas in the Hawaiian and northern Line Islands (Friedlander & DeMartini 2002; Sandin et al. 2008). Although belt transects provide more robust data than roving dives (because the survey area is standardized), they cover only small areas (e.g., 600 m^2^ in Sandin et al. [2008]) and are susceptible to biases associated with shark behavior (Ward-Paige et al. 2010b). More suitable are surveys dedicated to quantifying sharks and other large-bodied fishes over larger spatial extents (Richards et al. 2011). Results of one such effort (*n*= 80, 8000 m^2^ surveys) on the Great Barrier Reef (Robbins et al. 2006) suggest reef shark populations are considerably depleted on unprotected or lightly protected reefs compared with isolated reefs and reefs where all human activity is banned. Heupel et al.'s (2009) results are consistent with some of these findings and show reef shark catch per unit effort is higher in protected than in unprotected areas of the Great Barrier Reef.

**Figure 1:**
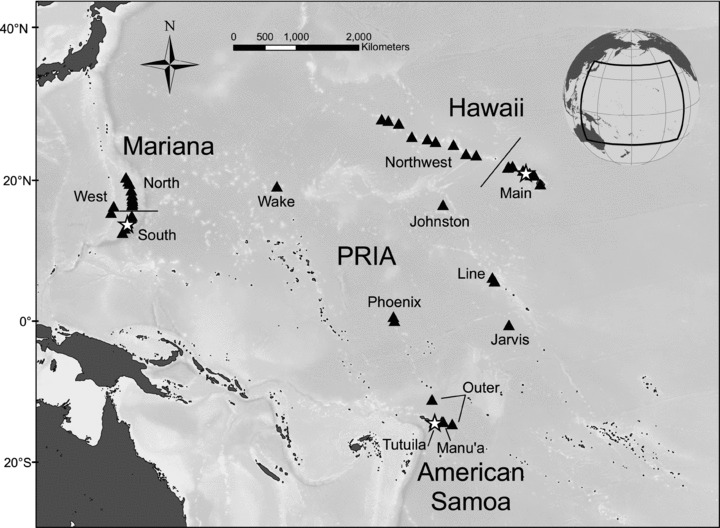
Reefs surveyed by towed divers 2004–2010 in the Pacific Ocean (triangles, survey reefs; white stars, large human population centers; PRIA, Pacific Remote Island Areas).

We tested whether the apparent negative effect of humans on shark densities holds after accounting for potentially important environmental factors. Scientific divers collected data on shark abundance during underwater surveys conducted between 2004 and 2010 around 46 U.S. Pacific islands (in an area 45° latitude by 58° longitude) as part of a National Oceanic and Atmospheric Administration standardized monitoring program (Fig. 1). This monitoring program includes some of the world's most isolated reefs, reefs near heavily populated areas, and reefs spanning a wide range of environmental conditions. Divers towed behind a boat collected data with a technique (towed-diver survey) developed specifically to survey large-bodied species of reef fishes (Richards et al. 2011).

We used these data to examine the effects of anthropogenic and environmental factors on reef shark abundances. We jointly, and in some cases individually, modeled the densities of 5 species of reef sharks: gray reef (*Carcharhinus amblyrhynchos*), whitetip reef (*Triaenodon obesus*), Galapagos (*C. galapagensis*), blacktip reef (*C. melanopterus*), and tawny nurse (*Nebrius ferrugineus*) sharks. We hypothesized that densities of reef sharks increase with primary productivity (potential increases in shark prey base), minimum sea surface temperature (SST) (physiological cold-tolerance limits in these warmwater species [Compagno 1984]), reef area (larger reefs may support disproportionally larger prey populations), and—in the case of whitetip reef sharks—reef physical complexity (species associated with rocks and crevices). We expected fishing (targeted, bycatch, recreational, illegal), anthropogenic depletion of prey and habitat changes, and pollution to negatively affect shark abundance; however, direct measures of such effects are scarce across the surveyed area. Therefore, we relied on a measure of human effects that was based on population size within a 200-km radius of each reef. To gain insights into the current status of Pacific reef sharks and estimate baseline densities, we used our models to predict the range of reef shark densities that might exist on these coral reefs in the absence of humans.

## Methods

### Study Area and Shark Densities

Between 2004 and 2010, divers recorded sharks observed on surveys conducted biennially around 46 individual U.S. islands, atolls, and banks (hereafter islands) in the central-western Pacific (Fig. 1). During each survey, a diver being towed behind a small boat recorded the identity and size of all fishes larger than 50 cm total length (nose to longest caudal fin lobe) encountered in a 10-m-wide belt (Richards et al. 2011). To ensure surveys represented a near-instantaneous snapshot, divers counted only individual fish in a 10 × 10 m area in front of them and were careful not to record the same fish more than once. All observers were experienced scientific divers with extensive training in fish identification. Divers were towed for 50 minutes on each survey at approximately 45 m/min, which is much faster than the swimming speed of divers conducting belt transects (typically 8 m/min). We used a global-positioning-system unit on the tow boat to calculate transect lengths. Average tow length was 2.2 km. Surveys followed fixed isobaths (generally 15–20 m depths) and were positioned evenly around an island, with the aim of covering most of the circumference of each island at the targeted isobaths (tows around small islands were closer to each other than those around large islands).

We analyzed only the towed-diver surveys that were conducted on forereefs (seaward slope of a reef) between 2004 and 2010 (*n*= 1607). We excluded earlier years (2000–2003) to limit the potential influence of a different set of observers, and abnormally short tows (length <1 km; *n*= 26) indicative of an anomalous dive. We also excluded surveys conducted in back reefs and lagoons (*n*= 152 surveys) because those reef types were not present at most islands surveyed.

Divers observed 8 shark species in the surveys. We first modeled an all-reef-sharks group, pooling the 5 shark species most closely associated with reefs (Table 1) to gain insight into the oceanographic, physical, and anthropogenic processes influencing this group of reef sharks at the basin extent. These species have a mostly tropical range (Compagno 1984) and have similar life histories (Smith et al. 1998) and diets (Randall 1977; Papastamatiou et al. 2006). Because all 5 species are larger than 50 cm at birth (Compagno 1984), juveniles of all species would have been recorded during surveys. We excluded tiger sharks (*Galeocerdo cuvier*) (*n*= 1) and hammerhead sharks (*Sphyrna lewini* and *S. mokarran*) (*n*= 90) from the analyses because these species are not restricted to reefs. We built separate models for the 2 most frequently encountered species, gray and whitetip reef sharks.

**Table 1:** Summary of towed-diver surveys, range of values of covariates, and total number of sharks observed by region*a*

						*Number of sharks observed*				
						
*Region*	*Reefs/surveys*	*Number of humans <200 km from reef (1000s)*	*Oceanic primary productivity (mg C·m^−2^·day^−1^)*	*Minimum monthly SST^b^ (°C)*	*Reef area (km^2^)*	*GR*	*WT*	*BT*	*Ga*	*Nu*
MHI	9/336	51–970	234–270	23.8–24.4	71–1662	16	27	−	12	−
NWHI	9/219	0–0.2	244–290	19.2–23.1	317–2447	62	102	−	104	−
Mariana I.	16/371	0–101	121–165	25.8–27.3	4–203	304	227	17	−	52
Am. Sam.	5/364	0.01–105	130–151	27.3–28.3	18–353	30	82	23	−	6
PRIA	7/317	0–0.01	147–445	25.3–27.3	20–240	2891	433	226	6	−
Total	46/1607					3303	871	266	122	58

a Abbreviations: MHI, main Hawaiian Islands; NWHI, northwestern Hawaiian Islands; Am. Sam., American Samoa; PRIA, Pacific remote island areas; GR, gray reef shark (C. amlyrhynchos); WT, whitetip reef shark (T. obesus); BT, blacktip reef shark (C. melanopterus); Ga, Galapagos shark (C. galapagensis); Nu, tawny nurse shark (N. ferugineus).

b Sea surface temperature.

### Environmental and Anthropogenic Covariates

We used remote-sensing data to examine the potential influences of oceanic productivity and SST on densities of reef sharks. We obtained mean oceanic primary productivity (mg C·m^−2^·day^−1^) between 1998 and 2007 from Aqua MODIS satellite monthly data combined in the vertically generalized production model (Behrenfeld & Falkowski 1997) at a spatial resolution of 0.083° (Oregon State University 2010) (Table 1 & Supporting Information). We obtained average monthly SST from AVHRR Pathfinder satellite data (1985–2006) for each source 4-km^2^ grid cell and calculated minimum temperature by selecting the lowest monthly average temperature per year and averaging these values across years (NOAA 2010) (Table 1 & Supporting Information). We also considered mean temperature, but it was highly correlated with minimum temperature (*r*= 0.97). For each surveyed island, we then computed a single value per covariate (mean primary productivity and minimum temperature) in ArcGIS 9.3 by taking its mean within a circle with a 50-km radius centered on the island after removing the 10 km closest to shore to avoid ocean-color distortion in shallow water.

We obtained the area of reef above 100 fathoms around each island from Rohmann et al. (2005) and used ArcGIS to supplement these data with values from bathymetric maps. Divers visually estimated reef complexity on a 6-point scale during their surveys (1, pavement or sand; 6, high and wide spurs and grooves).

We considered 3 measures of human effects on the basis of human population sizes (SEDAC 2010): distance to nearest population center, a metric of long-distance effects, with population center being the centroid of human population density within each region; humans per square kilometer of reef, a metric of local human effects, and humans within 200 km, a metric calculated by summing number of humans within a circle with a 200-km radius centered on each reef (combines local human population size with distance of human population to the reef) (Table 1). We chose 200 km as the radius of influence for the latter because it approximates the achievable range of a day trip by a typical fishing vessel on the basis of a traveling speed of 8–10 knots. We assumed human population was a reasonable measure of human effects in this region because most of the surveyed populated islands (including all population centers) have been settled for centuries, have broadly comparable levels of fisheries development (including widespread use of motorized boats and modern fishing gear) and reef fisheries with a mix of recreational, subsistence, and commercial fishing activities. A few of the remote islands (Midway, Wake, Johnston, and Palmyra) had large military bases in recent decades, but these are now either abandoned or, in the case of Wake and Midway, have only a small contingent of military personnel (see Williams et al. [2011] for more detail).

We did not include protection level in our analyses because the region's large marine protected areas were established only recently (e.g., 2006 in the northwestern Hawaiian Islands) and because protected areas cover only small percentages of the total coastline in populated areas (e.g., 5% around the main Hawaiian Islands). There is also some evidence that only areas that are strictly off limits to humans effectively protect reef sharks (Robbins et al. 2006). Moreover, in the larger, more isolated protected areas (e.g., northwestern Hawaiian Islands), remoteness rather than formal protection is probably the main factor limiting fishing because enforcement is generally light.

### Models of Shark Density

We modeled each shark group in a hierarchical Bayesian framework (Ntzoufras 2009) to take into account the nested nature of the data (i.e., tows nested within islands). Tow-level shark counts were modeled as a function of the area covered by each tow and the relevant island's mean shark density, which itself was modeled as a function of the covariates (details later). We considered covariates at the island rather than tow level because this is the geographic extent at which we expected them to influence shark abundance and because this aligns well with the mobility of the most common species in our study, gray reef sharks, which can cover dozens of kilometers daily (McKibben & Nelson 1986; Heupel et al. 2010). It is also difficult to directly pair data from towed-diver surveys with satellite oceanographic data given interference from bottom reflectance in shallow waters (Mumby et al. 2004). We standardized (centered and divided by standard deviation) covariates to aid model convergence.

Shark abundance data were negatively binomially distributed within individual islands. We therefore described the number of sharks per tow (*Y_t_*) as


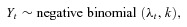
(1)

where *t* is individual tows and *k* is the overdispersion parameter. Mean numbers of sharks per tow (λ*_t_*) were related to the island-level mean shark density (*μ_i_*) and the number of hectares per tow as


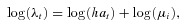
(2)

where *i* is individual islands. These island mean shark densities were nested within a normal hyperdistribution with mean values (*E_i_*) derived from a linear regression model


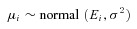
(3)


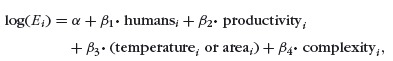
(4)

where α is the intercept and *β_j_* is the regression parameter corresponding to each covariate. We truncated the normal hyperdistribution to allow only positive shark density values because negative values cannot be used in the negative binomial distribution. The variance of this distribution (σ^2^) was weighted by sample size (i.e., tows per island). To define uninformative priors, we used normal distributions with a zero mean and variance of 100 for the regression coefficients and intercept, and a gamma distribution (with 0.01 for both parameters) for the hypervariance.

We conducted all hierarchical models in the Bayesian statistical software WinBUGS 1.4.3 (Supporting Information). We ran the Markov chain Monte Carlo (MCMC) algorithm 500,000 times with 2 chains. We discarded the first 100,000 runs as burn-in and calculated posterior quantities with remaining iterations. We used Gelman-Rubin diagnostics to test MCMC convergence (Ntzoufras 2009).

### Model Selection and Evaluation

A pairwise scatterplot and Pearson correlation coefficient matrix suggested that several covariates were sufficiently correlated to be problematic when modeled together (Graham 2003 & Supporting Information). The log values of the 3 human-population variables were highly correlated (pairwise *r* > 0.7), as was the log value of reef area with minimum temperature (*r*=–0.66; largely because Hawaii has several large islands in a cold-water region). We therefore first ran single-covariate models with each of the 3 human-population variables in which we used model weights to evaluate the support for each. Because reef area and minimum temperature relate to different ecological hypotheses, we modeled these confounded variables in 2 separate sets of models (Table 2). We also calculated variance inflation factors to verify that multicollinearity in our final models was low (variance inflation factors near or below 2) (Graham 2003).

**Table 2:** Posterior weights of selected models of shark density for each of the 3 modeled shark groups and the 2 sets of models, including the null and full models and all models with weights >0.05

	*Posterior weight^b^*		
	
*Model^a^*	*all species*	*gray reef shark*	*whitetip reef shark*
Set A			
Null	0	0	0.03
Prod	0	0	0.31
Prod + complexity	0	0	0.06
Human + prod	0	0	0.36^*^
Human + prod + complexity	0	0	0.07
Human + temp + complexity	0	0	0.07
Human + prod + temp	0.96^*^	0.94^*^	0.05
Human + prod + temp + complexity	0.04	0.06	0.01
Set B			
Null	0	0	0.03
Area	0	0	0.11
Human + area	0	0	0.18
Prod + area	0	0	0.10
Human + prod + area	0.98^*^	0.95^*^	0.19^*^
Human + prod + area + complexity	0.02	0.03	0.06

a Abbreviations: prod, primary productivity; human, log number of humans within 200 km; temp, minimum monthly temperature; complexity, complexity of reef structure; area, area of reef. Model set A includes the temperature variable, whereas model set B includes reef area.

b The model with the highest posterior weight in each case is indicated with an asterisk.

We selected the covariates to be included in our best models by calculating posterior model probabilities with latent indicator variables (*w_j_*) for each effect *j* (Ntzoufras 2009). These variables were defined as *w_j_*= 0 (variable exclusion) and *w_j_*= 1 (variable inclusion). Each *w_j_* had a Bernoulli(0.5) prior distribution to give both outcomes equal initial weights. We then used the MCMC history of these parameters to obtain the posterior probabilities of each variable by calculating the frequency with which each was included in the chain. Once a best model was identified, we removed these latent variables to estimate the value of the regression parameter (β*_j_*) and 95%CI. We added a step in each MCMC iteration to calculate predicted shark density in the absence of humans (i.e., the baseline) by setting human-effect variables to zero.

We assessed goodness of fit by generating a simulated data set of shark counts at every MCMC iteration, which measured the lack of fit of the simulated data set, and comparing this measure with the lack of fit of the original data set (Ntzoufras 2009). We used the sum of the squared residuals as the measure of lack of fit. We calculated the proportion of MCMC iterations in which the lack of fit measure was higher for the simulated data set than for the original data set to summarize the goodness of fit of the proposed model (Bayesian *p* -value). A value of approximately 0.5 indicates a good fit (Ntzoufras 2009).

## Results

In total 4620 sharks were observed, the majority of which were gray and whitetip reef sharks (71% and 19%, respectively) (Table 1). These were observed at almost all islands. Blacktip reef sharks (6%) were not observed in the Hawaiian Archipelago or at Johnston and Wake Atolls. Galapagos sharks (3%) were only observed at Johnston Atoll and in the Hawaiian Archipelago. Nurse sharks (1%) were observed mainly in the Mariana Archipelago.

### Shark Density Models

The WinBUGS model diagnostics showed clear model convergence (Supporting Information). Of the 3 variables pertaining to human effects, the log of humans per square kilometer of reef and the log of humans within 200 km were selected in almost every MCMC iteration (average *w_j_* > 0.999), but the latter yielded a better goodness of fit (i.e., Bayesian *p* value closer to 0.5). The log of distance to nearest population center had lower posterior weight (selected in fewer [65%] of iterations). We present model results for only the variable humans within 200 km because it performed best statistically and we believe it is conceptually better for this region than log of humans per kilometer of reef, which does not account for the effect of humans around unpopulated islands near large population centers.

Variable selection in both model sets (A with minimum temperature and B with reef area) followed a similar pattern for all reef sharks and gray reef sharks: models including humans within 200 km, primary productivity, and either minimum temperature (model set A) or reef area (model set B) had the highest posterior weight (>0.94) (Table 2). For whitetip reef sharks, the model with the highest weight in model subset A included only the effects of human population and primary productivity (weight = 0.36), not minimum temperature (Table 2). The reef-complexity variable had little weight (*w* < 0.25) in any of our models. Goodness of fit (Bayesian *p* value) for the best models of all 3 model sets were reasonably close to 0.5 (0.59 for model including temperature and 0.4 for model including reef area).

**Figure 2:**
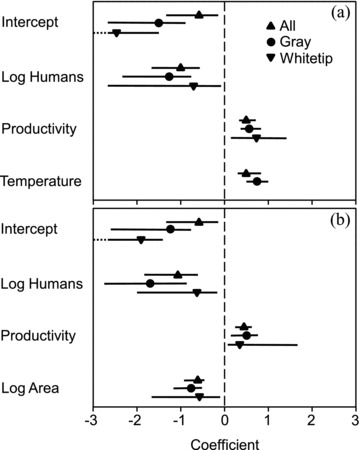
Parameter estimates (median and 95% credible interval of standardized coefficients) of the highest posterior weight model for 3 shark groups (all reef sharks, gray reef shark, whitetip reef shark) for the (a) sea-surface-temperature and (b) reef-area model sets (humans, number of humans < 200 km from reef; productivity, oceanic primary productivity; temperature, sea surface temperature; area, area of reef). Unstandardized parameter values are in Supporting Information.

**Figure 3:**
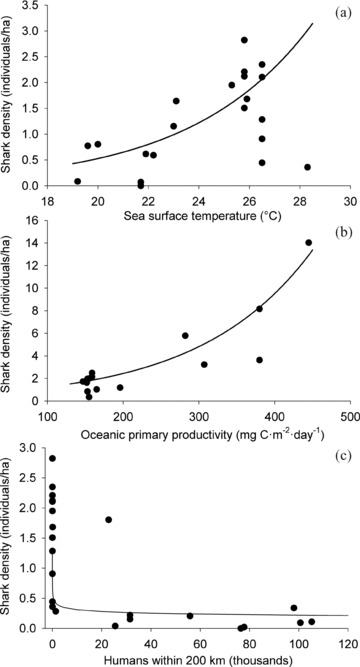
Relations between shark density and individual environmental covariates for islands with otherwise similar conditions: (a) temperature when human density is low (<100 humans within 200 km) and oceanic productivity is low (<300 mg C·m^−2^·day^−1^); (b) primary productivity when human density is low and water is warm (>25 °C sea surface temperature); (c) number of humans within 200 km when oceanic productivity is low and water is warm (black lines, expected shark density as a function of a single covariate obtained by keeping other covariates at a constant, representative, value for each group of islands).

In all cases, shark densities increased as oceanic primary productivity and minimum SST increased, but decreased as humans within 200 km and reef area increased (Fig. 2 & Supporting Information). To visualize these relations individually, we selected islands with similar environmental conditions (human population density, temperature, or productivity) and plotted their average shark densities with the expected model density under such conditions (Fig. 3). Densities of all reef sharks and gray reef sharks doubled for every 3.3 °C and 2.5 °C increase in minimum temperature (Fig. 3a) and for 100 and 90 mg C·m^−2^·day^−1^ increase in primary productivity (Fig. 3b), respectively. In comparison, whitetip reef sharks did not have a substantial response to temperature and were less influenced by primary productivity, doubling in density every 123 mg C·m^−2^·day^−1^. The effect of reef area on shark density followed a steeply declining power function for all 3 groups that leveled off at around 50 km^2^ (80% reduction in shark density).

The human effect on shark density had a similarly declining power function for all 3 groups of sharks; the strongest effect was on gray reef sharks (Figs. 2 & 3c & Supporting Information). Models indicated the initial number of humans within 200 km that was associated with a 20% decline in shark densities was <100 people for all 3-shark groups (Supporting Information). This effect leveled off at around 1000 humans within 200 km (approximately 60% reduction in shark density) and reached an approximately 90% reduction in shark density at very high human population densities (i.e., 1,000,000 humans) (Supporting Information).

**Figure 4:**
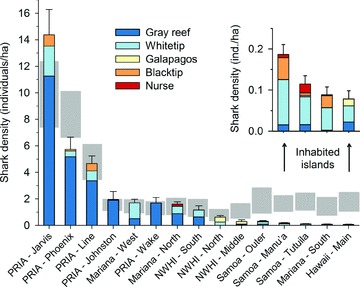
Mean (SE) observed densities of reef sharks in the U.S. Pacific (islands ordered from highest to lowest shark density; PRIA, Pacific remote island areas; NWHI, northwestern Hawaiian Islands; Samoa, American Samoa; gray rectangles, modeled 95% credible intervals of expected baseline shark density values given no humans within 200 km). Supporting Information contains a list of islands included in each region.

Simulated baseline densities for all reef sharks under the absence of humans were 1.1–2.4/ha for the main Hawaiian Islands, 1.2–2.4 for the inhabited islands of American Samoa, and 0.9–2.1 for inhabited islands in the Mariana Archipelago (Fig. 4 & Supporting Information).

## Discussion

Our results suggest humans now exert a stronger influence on the abundance of reef sharks than either habitat quality or oceanographic factors. All reefs near or within easy reach of highly populated islands had very low densities of reef sharks (approximately 0.1 sharks/ha), regardless of oceanic conditions (Fig. 4), whereas remote reefs, such as those around the Line and Phoenix Islands, supported much higher densities of reef sharks (approximately 7 sharks/ha). Accordingly, the size of human populations within 200 km was an important predictor in our models, with the presence of even very few people (<100) associated with a large decrease in densities of reef sharks (Supporting Information).

Possible explanations for the reduced abundance of reef sharks near human populations include fishing and a reduction of the sharks’ prey base. Although there are currently no active commercial reef shark fisheries around any U.S. Pacific island, reef sharks may be fished recreationally, taken incidentally, killed because they are perceived as a nuisance, and possibly taken illegally for their fins (Clarke 2004). Associated mortality levels are unknown, but even low levels of fishing mortality suffice to reduce abundances of reef sharks (Robbins et al. 2006; Ferretti et al. 2010; Ward-Paige et al. 2010a), and sharks released from incidental captures may still die as a result of stress or physical trauma (Mandelman & Skomal 2009). In addition, reef sharks depend on coral reef fishes for over 70% of their diet (Randall 1977; Papastamatiou et al. 2006), and humans are reducing this resource through direct exploitation of prey fish and by changing essential fish habitat (Williams et al. 2011).

As expected, our models indicated reef shark abundance is also substantially influenced by oceanic primary productivity. Isolated reefs located in warm waters with the highest productivity had the highest shark densities (e.g., up to 14 sharks/ha around Jarvis Island). In contrast, isolated reefs in warm but relatively unproductive waters (e.g., Wake Atoll, northern Mariana Islands) had fewer reef sharks (around 1.7 sharks/ha). The positive influence of primary productivity on marine animal production has been established theoretically (Jennings et al. 2008) and empirically (Chassot et al. 2010) and is likely mediated by bottom-up increases in the density of shark prey (Bakun 1996).

The influence of other environmental factors on reef shark densities, and their underlying mechanisms, are less clear. Lower minimum SSTs reduce metabolic rates and may thus affect population densities through effects on growth and reproduction ( Jennings et al. 2008). Alternatively, lower SST may affect shark densities through species–specific, physiologically driven effects. Decreases in gray reef shark and increases in Galapagos shark densities along an increasing latitudinal gradient in the northwestern Hawaiian Islands (Supporting Information) suggest such effects. Gray reef sharks may approach the limit of their cold-water tolerance in the northernmost reefs given their lower latitudinal range (approximately 30° N, whereas Galapagos shark range is approximately 36° N) (Compagno 1984). The high correlation between reef area and temperature in our data set (*r*=–0.66), however, complicates interpretation of their effect on shark densities because it is impossible to separate their effects (i.e., best models for all reef sharks and gray reef sharks included either temperature or reef area). Contrary to expectations, shark densities were higher at smaller reefs. Such patterns may arise for several reasons. First, reef area was positively correlated with human-effect variables (*r*∼ 0.22) because larger islands tended to be the most heavily populated. Second, small, isolated islands are often the only available shallow habitat in large ocean expanses and may thus attract transient reef sharks. Movement patterns of reef sharks are poorly understood, but gray reef sharks, for example, are capable of traversing large (>120 km) stretches of open water (Heupel et al. 2010). Finally, deepwater habitat is often closer to survey depths around smaller islands (i.e., steeper slopes) and reef sharks may be present there in greater densities during daytime (Chapman et al. 2007).

As with all shark studies that are based on observations by divers, the surveys we modeled only quantified the density of sharks present at easily accessible depths (10–25 m) and times (daylight hours), not of the entire shark community. Peak densities of reef sharks may occur at night (Chapman et al. 2007) or at depths greater than those typically surveyed by divers (e.g., 30–40 m according to Papastamatiou et al. [2006]). Data from hook-and-line surveys in the Hawaiian archipelago reveal the presence of a primarily deeper-water species (sandbar shark [*C. plumbeus*]) (Papastamatiou et al. 2006) not encountered in any of our surveys. Bottom-associated species (i.e., whitetip and nurse sharks) also are harder to observe, especially in high-relief areas, and may be somewhat underrepresented in our samples. Such sampling biases should be similar across regions, however, so that the patterns of reef shark density in our analyses should be valid in at least relative terms.

One important difference between our results and those of previous studies in the region is the use of data from towed-diver surveys (Richards et al. 2011), which generated reef shark densities significantly lower than those from small-scale belt transects (Friedlander & DeMartini 2002; Sandin et al. 2008) or stationary point counts (Williams et al. 2011). For example, estimated reef shark densities for Palmyra Atoll and Kingman Reef were 3.4 sharks/ha and 6.8 sharks/ha, respectively in towed surveys, whereas belt surveys of the same reefs resulted in 50 sharks/ha and 170 sharks/ha, respectively (as calculated from the shark biomasses reported in Sandin et al. [2008]). Mobility of these predators likely introduces positive bias (i.e., overestimation) in the latter noninstantaneous surveys at small spatial extents (Ward-Paige et al. 2010b; Dickens et al. 2011). Towed-diver surveys reduce (but cannot eliminate) this positive bias by surveying a larger area of reef per survey and by quickly (45 m/min) moving divers into new areas to prevent sharks aggregating around the surveyors (Richards et al. 2011). These spatially extensive surveys therefore likely reflect shark densities more accurately than more commonly employed survey techniques at small spatial extents.

Although biased estimates of shark density may be of little consequence when they are used only to compare relative differences in shark populations (e.g., across space or time), such biases are important when shark abundance (or biomass) estimates are compared with other components of the reef fish community. The very high shark densities reported in Sandin et al. (2008), for example, resulted in estimated top predator biomass (mainly sharks) being higher than that of each of the lower trophic groups (i.e., carnivores, planktivores, and herbivores) and led to the suggestion that remote coral reefs accommodate inverted biomass pyramids. Our empirical results, as well as those from simulation studies (Ward-Paige et al. 2010b), instead suggest that apparent inverted biomass pyramids are artifacts of sampling biases associated with surveys at small spatial extents.

### Modeled Quantitative Baselines

Our simulation of baseline shark densities, combined with other recent studies (Robbins et al. 2006; Graham et al. 2010; Ward-Paige et al. 2010a), support the conclusion that in the absence of humans sharks would be a conspicuous presence on coral reefs. For American Samoa, for example, our simulation estimated baseline densities between 1.2 sharks/ha and 2.4 sharks/ha, which suggest current densities are at 4–8% of their baseline. The inhabited islands in Hawaii and the Mariana Archipelagoes show similarly low reef shark densities (3–7% and 4–10% of baseline values, respectively). Our baseline estimates account for differences in environmental conditions between populated and isolated reefs; thus, we avoided a common pitfall in studies that use remote locations with distinct environmental conditions to infer pristine conditions. Although our baseline estimates may provide an impetus for shark conservation, they are likely to be less useful for setting specific management targets (Marsh et al. 2005) until ecological and physical controls on carrying capacity are better understood.

The absence of sharks and other large predators on coral reefs influenced by humans may affect these ecosystems via trophic cascades (Stevens et al. 2000; Myers et al. 2007), prey behavioral changes (Ferretti et al. 2010), and increased community susceptibility to perturbations (Bascompte et al. 2005). Increasing abundances of reef sharks around populated islands would likely require a concerted ecosystem-level effort aimed at reducing exploitation of both sharks and their prey and identifying and protecting critical habitats. The main factor currently sustaining the high reef shark densities recorded around some islands appears to be geographic isolation. The recent implementation of marine national monuments at most isolated U.S. Pacific islands may substantially increase the probability of persistence of reef shark populations, but effective enforcement and additional fishing regulations elsewhere would also be necessary to slow the decline of these species (Hoffmann et al. 2010).
