# Supplementary material for: Re-Creating Missing Population Baselines for Pacific Reef Sharks
Source: Conserv Biol. 2012 Jun;26(3):493–503. doi: 10.1111/j.1523-1739.2012.01835.x (PMC3494310; doi:10.1111/j.1523-1739.2012.01835.x)
Supplement: Supplementary file 1 [file cobi0026-0493-SD1.doc]

Appendix S1 – Oceanic primary productivity derived from satellite imagery (average from 1999 – 2009).


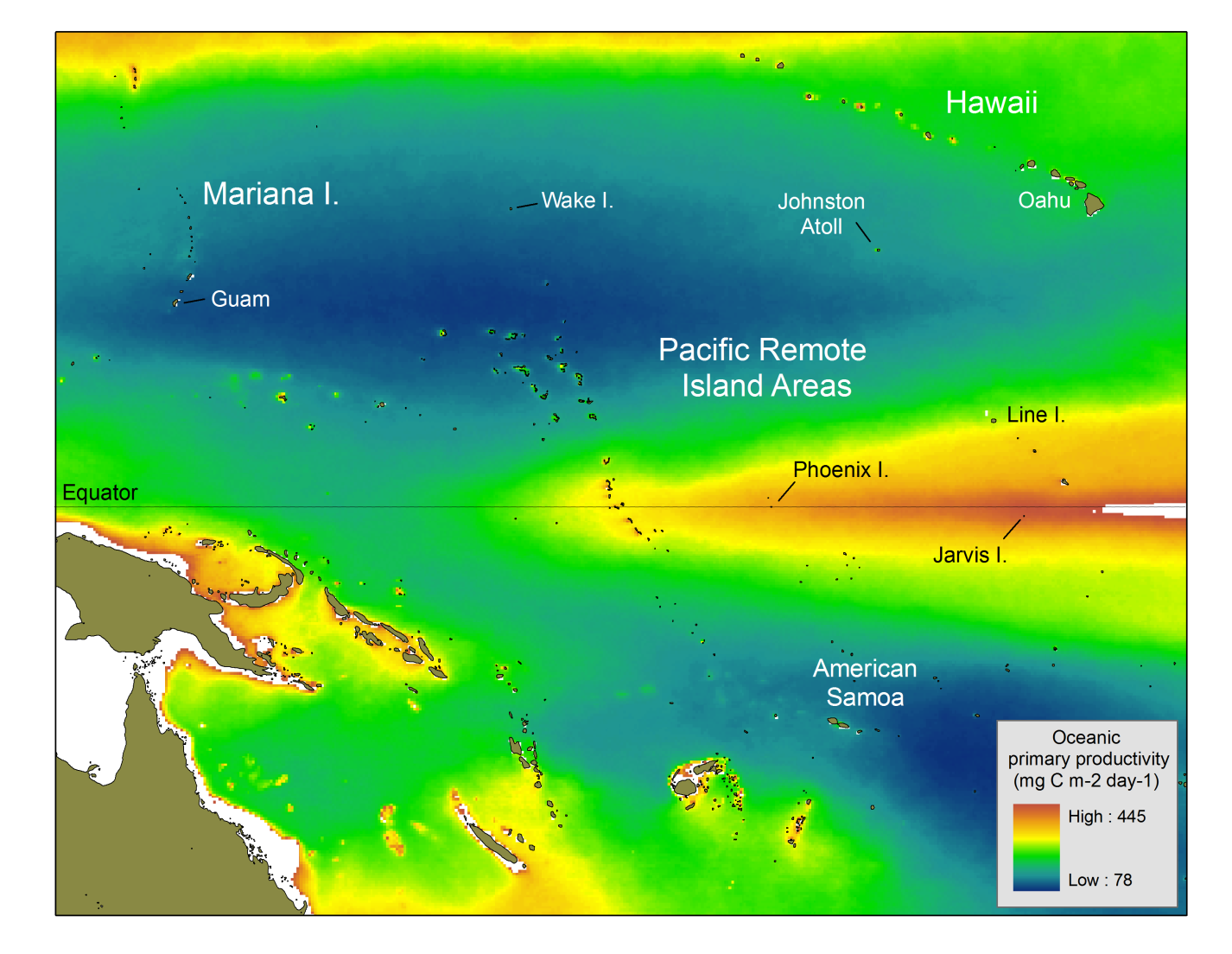


Appendix S2 – Minimum mean monthly sea surface temperature derived from satellite imagery (average from 1985 – 2008).


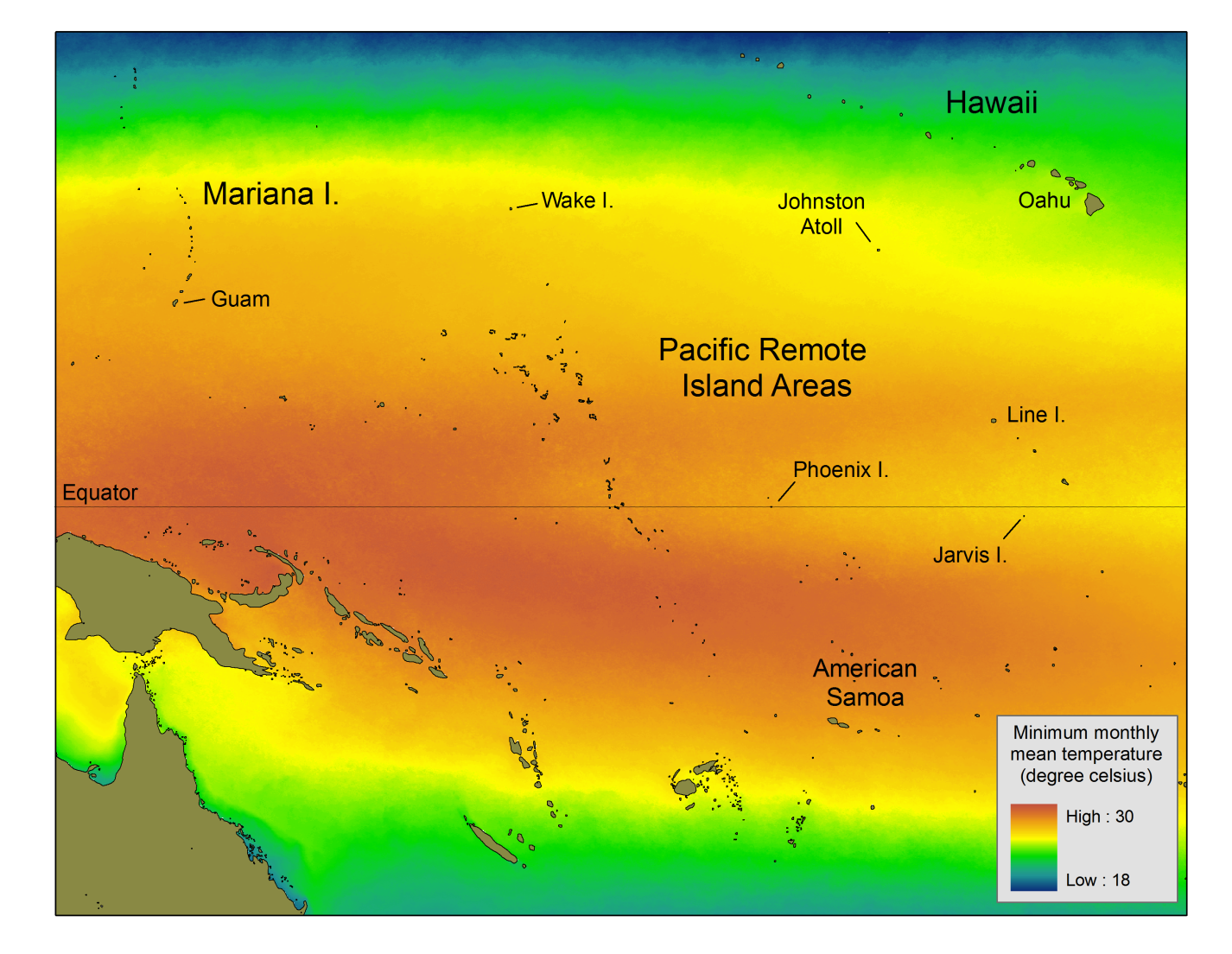


Appendix S3 – WinBUGS code and convergence diagnostics for the selected model (reef shark group dataset - full model; diagnostics for gray reef and whitetip reef shark models were similar).


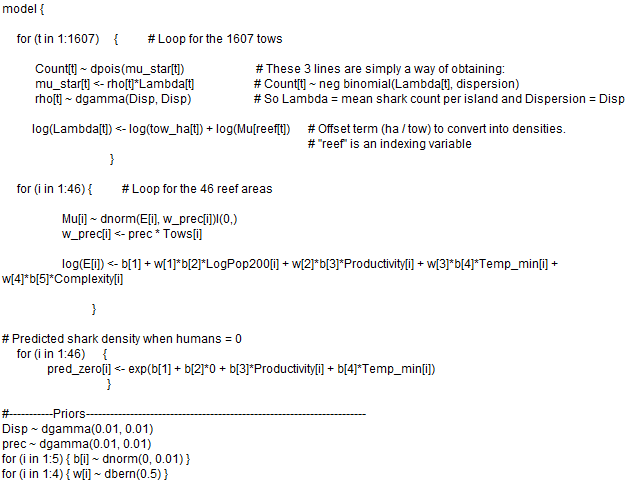


Gelman-Rubin convergence statistic for b values


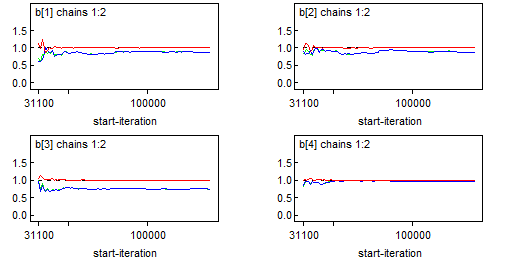


Kernel density estimates (for b values)


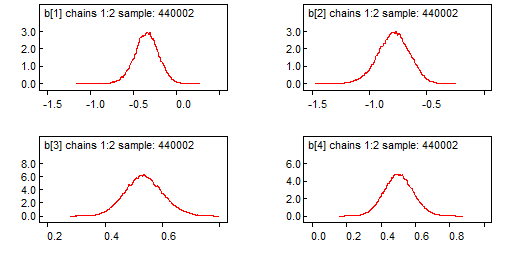


Appendix S4 – Modeled effect of number of humans within 200 km on expected reef shark densities (all-species combined).

Appendix S5 – Expected reef shark density (all species; given presence of shallow hard-bottom habitat) in the absence of human impacts as derived by the statistical model presented in the
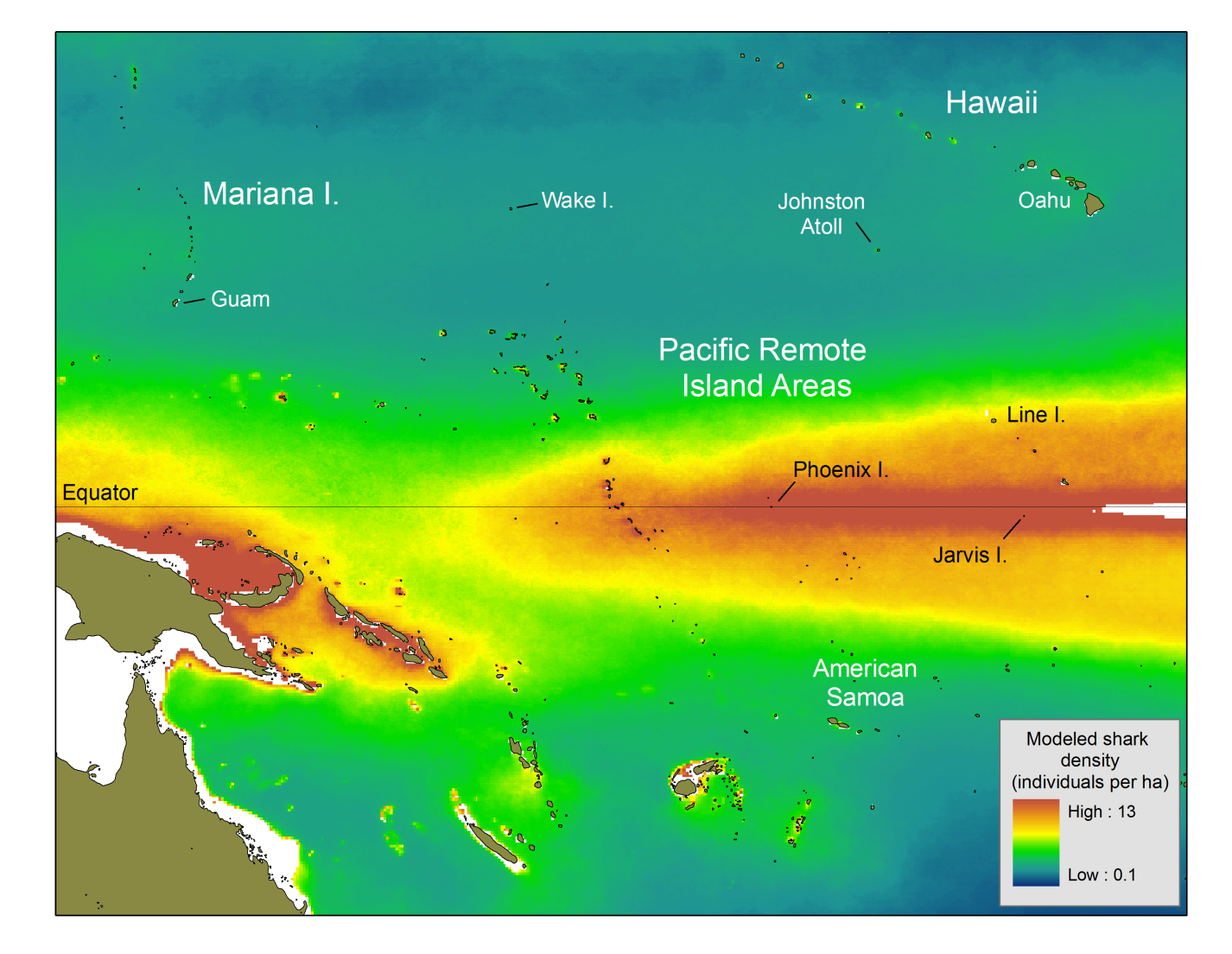
current study (humans + primary productivity + minimum temperature model).

Appendix S6 – Reef shark density in the Northwestern Hawaiian Islands (NWHI) by region. Galapagos shark = gray, Gray reef shark = black. South = French Frigate Shoals, Necker Island, Gardner Pinnacles; Middle = Maro Reef, Lisianski Island, Laysan Island; North = Pearl and Hermes Atoll, Midway Atoll, Kure Atoll.

Appendix S7 – Correlation between explanatory variables (Pearson’s correlation coefficient). Values above 0.5 are in bold.

|  | Ln humans per km^2^ of reef | Ln distance to  human population center | Ln human population  < 200 km | Ln reef area above  100 fathoms | Mean oceanic  primary productivity |
| --- | --- | --- | --- | --- | --- |
| Ln dist. to  human pop. center | **- 0.68** |  |  |  |  |
| Ln human pop. within 200 km | **0.68** | **-0.77** |  |  |  |
| Ln reef area above  100 fathoms | 0.20 | -0.02 | 0.23 |  |  |
| Mean oceanic  primary productivity | - 0.28 | 0.37 | - 0.27 | 0.39 |  |
| Minimum monthly  SST | 0.25 | -0.47 | 0.09 | **- 0.66** | - 0.46 |

Appendix S8 - Median parameter values, with 95% credible interval, for models with highest weight, in the 3 datasets tested (all species combined, gray reef sharks only, whitetip reef sharks only), for the 2 model sets. Parameter values are for original, un-standardized, data.

| Parameter | Dataset | 2.50% | Median | 97.50% |
| --- | --- | --- | --- | --- |
| Intercept | All species | 0.1 | 1.0 | 1.9 |
|  | Gray^a^ | -0.63 | 0.57 | 1.95 |
|  | Whitetip^b^ | -1.44 | -0.19 | 0.88 |
| Log Humans^c^ | All species | -0.33 | -0.19 | -0.12 |
|  | Gray | -0.57 | -0.28 | -0.13 |
|  | Whitetip | -0.34 | -0.12 | -0.001 |
| Productivity^d^ | All species | 0.0038 | 0.0057 | 0.0077 |
|  | Gray | 0.0039 | 0.0066 | 0.0092 |
|  | Whitetip | 0.0004 | 0.0033 | 0.0066 |
| Log Area^e^ | All species | -0.47 | -0.35 | -0.25 |
|  | Gray | -0.55 | -0.40 | -0.26 |
|  | Whitetip | -0.68 | -0.34 | -0.14 |

| Parameter | Dataset | 2.50% | Median | 97.50% |
| --- | --- | --- | --- | --- |
| Intercept | All species | -7.9 | -6.2 | -4.2 |
|  | Gray | -10.2 | -8.6 | -6.4 |
|  | Whitetip | -3.1 | -2.3 | -1.0 |
| Log Humans | All species | -0.21 | -0.15 | -0.10 |
|  | Gray | -0.28 | -0.18 | -0.11 |
|  | Whitetip | -0.21 | -0.12 | -0.04 |
| Productivity | All species | 0.0052 | 0.0068 | 0.0086 |
|  | Gray | 0.0057 | 0.0077 | 0.0100 |
|  | Whitetip | 0.0016 | 0.0056 | 0.0077 |
| Temperature^f^ | All species | 0.13 | 0.21 | 0.27 |
|  | Gray | 0.20 | 0.28 | 0.34 |
| ^a^ Gray reef shark – *C. amblyrhynchos*, ^b^ whitetip reef shark – *T. obesus*, ^c^ humans <200 km, | | | | |
| ^d^ primary productivity, ^e^ log human within 200 km, ^f^ minimum monthly sea surface | | | | |
